# Early signals of water limitations begin at the root–soil interface: linking rhizosphere drying to water uptake decline

**DOI:** 10.1111/nph.70879

**Published:** 2025-12-26

**Authors:** Sara Di Bert, Pascal Benard, Rong Jia, Fabian J. P. Wankmüller, Seren Azad, Anders Kaestner, Andrea Nardini, Timothy J. Brodribb, Andrea Carminati

**Affiliations:** ^1^ Department of Environmental System Science ETH Zurich Zürich 8092 Switzerland; ^2^ Department of Soil and Environment SLU Uppsala 75007 Sweden; ^3^ State Key Laboratory of Maize Bio‐breeding, College of Agronomy and Biotechnology China Agricultural University Beijing 100193 China; ^4^ PSI Center for Neutron and Muon Sciences Villigen PSI 5232 Switzerland; ^5^ Department of Life Sciences University of Trieste Trieste 34127 Italy; ^6^ School of Natural Sciences University of Tasmania Hobart TAS 7001 Australia

**Keywords:** neutron radiography, rhizosphere hydraulics, root water uptake, soil texture, stomatal regulation

## Abstract

- Understanding when and where drought stress originates in the soil–plant continuum is essential for predicting plant responses to climate change. While stomatal closure is a well‐known reaction to declining soil moisture, the precise hydraulic trigger remains unresolved. We investigated whether the initial reduction in root water uptake is concomitant with a localized depletion of water near the root surface.
- Using high‐resolution neutron radiography, we visualized dynamic changes in water distribution near maize (*Zea mays* L.) roots under controlled drying. We quantified the shift in water uptake patterns and their impact on whole‐plant water use.
- Under wet conditions, roots primarily extracted water from the bulk soil. As soil moisture declined below a texture‐dependent threshold, hydraulic conductivity dropped, preventing water flow from the bulk soil into the rhizosphere. This caused a shift in water uptake to the rhizosphere, coinciding with reduced transpiration and stomatal downregulation. The transition occurred *c*. −5 kPa in sandy soils and −200 kPa in loamy soils.
- These results provide direct evidence that an early hydraulic limitation during soil drying occurs in the rhizosphere, particularly in sandy soils. This redefines the rhizosphere as a dynamic control zone that mediates early drought responses and links microscale hydraulic behavior with whole‐plant function.

## Introduction

The capacity of plant roots to extract water from the soil is central to plant performance and ecosystem function, especially under drought conditions. Across terrestrial biomes, intensifying water limitation – rather than energy or nutrient scarcity – is increasingly projected to be the dominant constraint on vegetation productivity (Dai, 2013; Allen *et al*., 2015; Fu *et al*., 2022). A well‐established plant response to progressive soil drying is the decline in stomatal conductance, which restricts transpiration and photosynthetic carbon assimilation. While this physiological outcome is well documented, the factors that cause it are still being debated.

Stomatal closure follows a decline in leaf water potential and consequently in leaf cell turgidity, which then triggers stomatal closure via passive and active signals (Buckley, 2005; McAdam & Brodribb, 2014). Less clear is what is the primary cause of the decline in leaf water potential. The prevailing view is that losses in leaf water potential are caused by a loss in hydraulic conductance within plant tissues – including leaves, xylem, and root cortical layers – each of which responds to water stress through distinct mechanisms (Sperry *et al*., 2002; Sack & Holbrook, 2006; Martin‐StPaul *et al*., 2017; Rodriguez‐Dominguez & Brodribb, 2020; Bourbia *et al*., 2021). Under drought conditions, hydraulic conductance declines primarily in leaves and roots – the two main resistive organs in the plant hydraulic pathway – due to both within‐xylem limitations, such as embolism formation caused by cavitation, and outside‐xylem limitations, including turgor loss and mesophyll collapse in leaves and increased suberization or aquaporin downregulation in roots (North & Nobel, 2000).

However, an alternative hypothesis is that the primary constraint to water transport during drought arises not within the plant, but in the soil. As soils dry, the decline in soil water potential and unsaturated hydraulic conductance leads to an increased resistance near the root surface, where steep water potential gradients are formed (Gardner, 1960; Passioura, 1988; Carminati *et al*., 2010; Carminati & Javaux, 2020). These external limitations can precede internal hydraulic failure, with the resulting water potential gradients transmitted through the plant to the leaves. Consequently, a significant drop in leaf water potential may occur before any decline in plant hydraulic conductance.

Empirical and modeling studies increasingly support this view. Several studies have shown that reductions in water uptake from soil often precede significant reductions in leaf or xylem hydraulic conductance (Rodriguez‐Dominguez & Brodribb, 2020; Abdalla *et al*., 2022). These findings suggest that a decline in soil water supply – not internal damage – may be the onset of plant water stress and drought response.

**Fig. 1:**
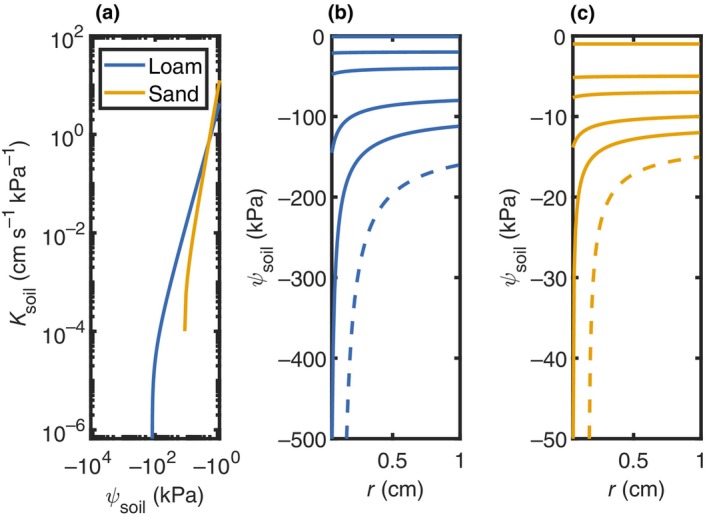
Soil hydraulic conductance and soil water potential gradients toward roots surface. (a) Hydraulic conductance ($K_{\text{soil}}$) vs soil water potential ($\psi_{\text{soil}}$) for loam (blue) and sand (orange) assuming constant transpiration rate and illustrating the asymptotic loss of conductance at a critical soil water potential (i.e. when the curves drop vertically). (b, c) Radial $\psi_{\text{soil}}$ profiles toward root surface (*r*) for loam (b) and sand (c). Solid lines show the development of water potential gradients near the root surface as soil dries. Dashed lines indicate conditions where gradients become too steep and water cannot reach the root surface at the imposed rate. This forces the plant to extract water primarily from the rhizosphere and its own tissues, with reduced flow from the bulk soil, eventually leading to stomatal closure to reduce transpiration and the imposed rate of root water uptake.

The biophysical basis for this soil limitation has long been explained. The pioneering work by Richards (Richards, 1928) and Gardner (Gardner, 1960) distinguished two essential components of plant water relations: the ability of roots to absorb water and the movement of water through unsaturated soil toward the root. As soils dry, their unsaturated hydraulic conductivity drops by orders of magnitude (Mualem, 1976; van Genuchten, 1980; Fig. 1a), inducing steep water potential gradients near the root–soil interface (Draye *et al*., 2010; Fig. 1b,c, solid lines). Eventually, there is a critical soil water potential when water can no longer reach the root surface at the rates demanded by the atmosphere and (maximum) canopy conductance (Fig. 1b,c, dashed line). This critical condition is, among others, specific to soil conditions such as texture: for example, it is reached at less negative soil water potentials in sandy soils due to their steep conductivity curve. As a result, roots increasingly depend on a progressively thinner film of moisture in the rhizosphere, the narrow zone of soil directly adjacent to the root surface (dashed lines in Fig. 1b,c) (Carminati *et al*., 2010). At the same time, the water potential at the root–soil interface, and consequently in the leaves, rapidly drops.

Although the idea that soil acts as the primary constraint on water transport during drought has been adopted to predict stomatal behavior across scales, from the rhizosphere to the plant and canopy level (Wankmüller *et al*., 2024), direct empirical evidence of it remains elusive. The challenge remains much as Wadleigh & Richards (2013) described: progress in understanding root–soil water dynamics has been hindered by the difficulty of resolving moisture gradients in the immediate vicinity of roots.

Neutron radiography offers a powerful tool to observe water dynamics in soil and roots. Because hydrogen strongly attenuates neutrons, this technique enables noninvasive, high‐resolution imaging of water content in soil and plant tissues (Oswald *et al*., 2008; Cai *et al*., 2022). Previous studies using neutron imaging have revealed that the rhizosphere has different water holding capacity compared with the surrounding bulk soil. Moradi *et al*. (2011) showed that the rhizosphere retains more water than the bulk soil during drying. The enhanced water retention of the rhizosphere arises from a combination of factors: root exudates, particularly mucilage and other extracellular polymeric substances (EPS) exuded by microbial communities, contribute to the formation of a cohesive, hydrated microenvironment (Benard *et al*., 2019a). They alter the soil's hydraulic properties by increasing water retention and reducing evaporation while root growth impacts pore size distribution (Lucas *et al*., 2019). These features might buffer the root against desiccation and might delay the onset of steep water potential gradients during early stages of soil drying. Yet, no study has directly linked these rhizosphere‐scale water dynamics to whole‐plant transpiration responses under drying conditions – leaving a critical gap in our understanding of how microscale hydraulic properties propagate to influence macro‐scale plant function.

We hypothesize that as soils dry below critical soil water potentials ($\psi_{\text{crit}}$(MPa)), the flow of water in the soil cannot match the transpiration demand. Hence, water extraction becomes confined to the rhizosphere and root cortical tissues, inducing a rapid decline in leaf water potential and consequently triggering stomatal closure.

This sequence of events has never been clearly observed. By means of high‐resolution neutron radiography, we aimed to capture the spatiotemporal dynamics of water depletion around maize roots grown in soils of contrasting textures. By identifying when and where water uptake becomes restricted during progressive drying, we aimed to determine whether rhizosphere‐scale hydraulic limitation governs the initiation of stomata closure.

## Materials and Methods

### Plants and soil preparation

Maize seedlings (*Zea mays* L. B73) were grown in aluminum rhizoboxes – narrow soil‐filled containers (10 × 12 × 1 cm) designed for root observation – filled with either sandy or loamy soil as used and defined in Vetterlein *et al*. (2021). To ensure uniformity, the soils were sieved to a particle size of < 2 mm before filling. The rhizoboxes were designed with detachable faces, allowing for controlled soil deposition. Soil was poured into each container through a 2‐mm sieve while the containers were positioned horizontally to achieve homogeneous packing visible in the image plane and minimize layering effects. Once filled, the detachable faces were sealed, and the containers were carefully repositioned to a vertical orientation. This procedure resulted in an average dry bulk density of 1.88 g cm^−3^ for sandy soil and 1.56 g cm^−3^ for loamy soil.

Maize seeds were germinated in darkness on moist filter paper for 3 d before transplanting. Seedlings were planted at 1 cm depth in the soil‐filled containers and grown under controlled environmental conditions: with a 14 h photoperiod, light intensity of 300 μmol m^−2^ s^−1^, day : night temperatures of 24°C : 19°C, and a relative humidity of 60%. A liquid fertilizer solution (PlantAktiv, Hauert HBG Dünger AG, Grossaffoltern, Switzerland) was applied at the start of the experiment to ensure adequate nutrient availability.

The volumetric soil water content was maintained at 18% for sandy soil and 20% for loamy soil, with irrigation provided daily for the first 3 d, followed by every second day thereafter. Plants were 14 d old at the initiation of the neutron radiography experiment.

### Time‐series neutron radiography

To visualize spatial and temporal root water uptake (RWU) dynamics, we employed neutron radiography, a high‐sensitivity imaging technique that enables noninvasive tracking of water‐containing materials in soil (Oswald *et al*., 2008; Moradi *et al*., 2009; Perfect *et al*., 2014; Cai *et al*., 2022). The high resolution of neutron imaging allows the clear distinction of roots from the surrounding soil (see Supporting Information Fig. S1).

Neutron radiography was performed on a total of 11 maize plants per soil type (*n* = 11 for sand, *n* = 11 for loam) over three beamtime sessions: two at the ICON beamline and one at the NEUTRA beamline at the Paul Scherrer Institute (PSI), Villigen, Switzerland. A charge‐coupled device (CCD) camera detector with a 2048 × 2048‐pixel array was used, providing a 150 × 150‐mm field of view and a pixel size of *c*. 70 μm.

To monitor plant water dynamics under both transpiring and nontranspiring conditions, we controlled illumination within the imaging station to simulate natural day and night cycles. A high‐intensity LED lamp, calibrated to match the plants' previous growth conditions, was positioned above the samples. When the light was on, mimicking daytime conditions, plants were expected to transpire. During this phase, they were continuously scanned over extended periods to monitor water depletion and RWU dynamics throughout the drying process. By contrast, when the light was off, simulating nighttime conditions, transpiration was assumed to cease, allowing the plants to reach equilibrium in water potential. Under these stable conditions, measurements were taken at discrete time points, averaging five radiographs per sample, as no further changes in water distribution were expected. During the experiments, the soil was covered with tape to ensure that the soil water depletion was due to transpiration only. Inside of the beamline, the average temperature was 26°C and relative humidity was 55%.

### Image processing

Neutron radiographs were postprocessed to correct for imaging artifacts and enhance accuracy in water content quantification. A comprehensive correction procedure based on the method described by Carminati *et al*. (2019) was applied, which includes flat‐field correction (radiographs taken without the sample), dark‐current correction (signal recorded by the camera in the absence of a neutron beam), and correction for scattered neutrons. This integrated approach accounts for systematic variations in detector response and minimizes artifacts caused by neutron scattering.

The neutron attenuation coefficients of aluminum and dry soil were determined using radiographs of a reference rhizobox filled with dry soil. After applying a logarithmic transformation to convert transmission data into optical thickness, the contributions from aluminum and dry soil were subtracted. This isolated the optical thickness due to water, enabling precise quantification of water content in the sample.

All image processing and data analysis were performed using imagej2 (Rueden *et al*., 2017, v.1.54f) and matlab (The MathWorks Inc., 2021, version 2021a).

### Segmentation and water content analysis

To investigate potential temporal differences in water dynamics between soil compartments, we aimed to separately quantify water content changes in the bulk soil (defined as soil distant from roots) and the rhizosphere (defined here as the soil near the root, including the root itself). Radiographic images were segmented using a multi‐step image processing workflow. Initial preprocessing involved denoising the radiographs using a combination of median filtering and nonlocal means filtering. Following denoising, a dynamic contrast adjustment was applied to enhance the visibility of structural features.

**Fig. 2:**
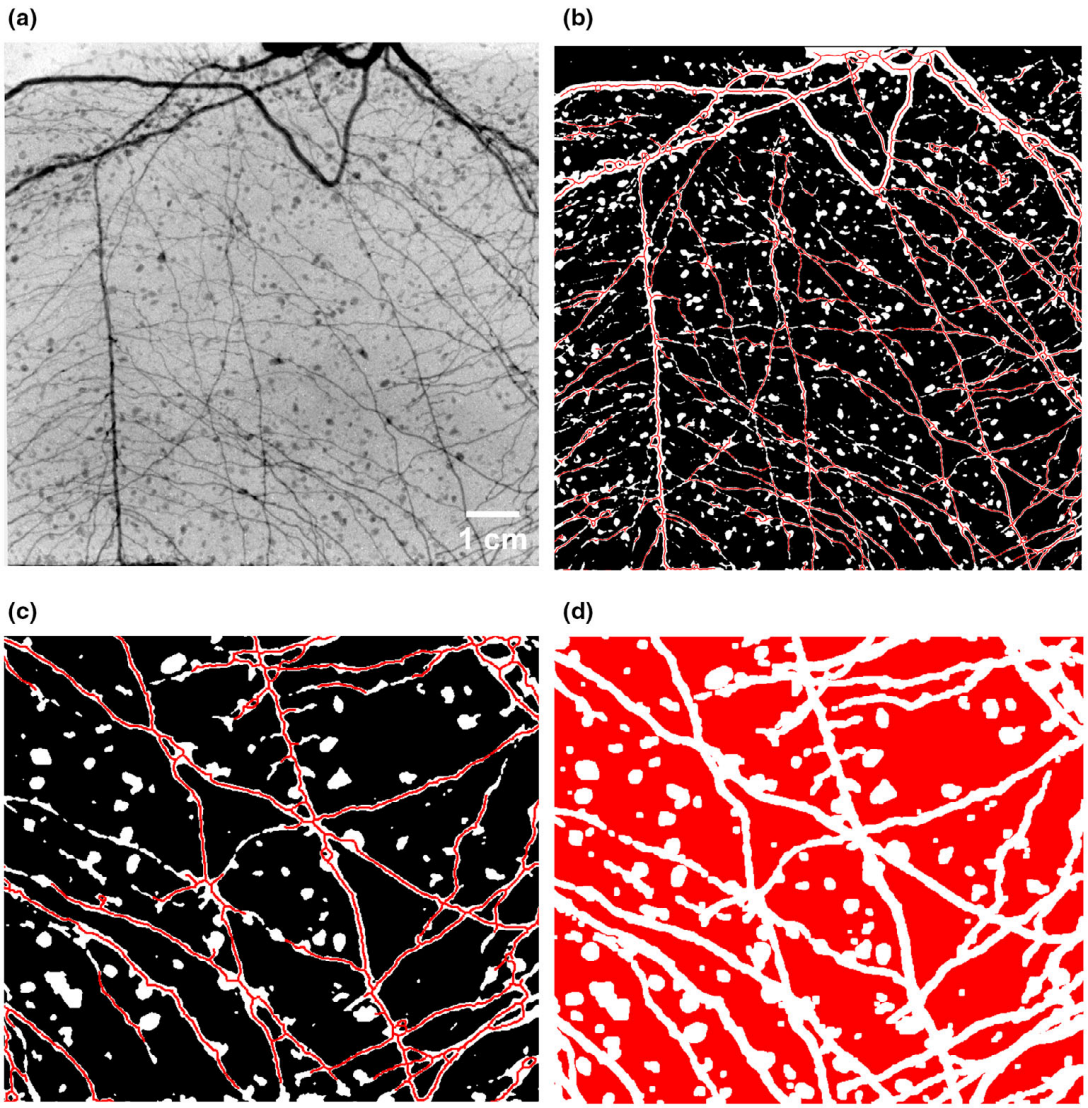
Image segmentation workflow. Images show *Zea mays* L. roots. (a) Original radiograph. (b) Segmented and skeletonized image, with the rhizosphere region highlighted in red. (c) Enlarged view of segmented and skeletonized image. (d) Enlarged view of dilated image where the bulk soil is marked in red.

To define the rhizosphere and bulk soil regions, root skeletons were extracted to represent the central axis of RWU. For loam, where image contrast was lower, root skeletons were manually delineated to ensure accuracy. For sand, which offered higher contrast, an automated segmentation workflow – combining adaptive thresholding and morphological operations – was used to isolate root structures, followed by skeletonization to extract their centerlines and exclude remaining soil aggregates. In both cases, the skeleton itself was used to define the rhizosphere, capturing only the pixels most directly aligned with root positions. The bulk soil was defined by dilating this skeleton mask sufficiently to exclude any root‐associated pixels, ensuring that only soil distant from the root was included (Fig. 2).

Because neutron radiography provides 2D projections through the sample thickness (*c*. 1 cm), the skeletonized root pixels effectively represent not just the root itself but also its projection along the beam path. Consequently, the region referred to as rhizosphere includes the root together with the adjacent soil captured within this *c*. 1 cm projection, comprising the soil in front of and behind the root.

This approach does not yield the actual water content in the rhizosphere, which was found to change gradually toward the root surface (Moradi *et al*., 2011). However, it allows for exploring the temporal dynamics of the relative changes in water content close and far from the root.

To approximate soil water potential as experienced by the plant, we converted measured water content to water potential using the water retention curve from Vetterlein *et al*. (2021). While these values are approximate and not absolute, they provide a meaningful reference for assessing relative changes in soil dryness over time.

### Root water uptake and water content dynamics

To evaluate how water content in the rhizosphere changes relative to the bulk soil over time, we analyzed the ratio of their temporal water content changes, expressed as $\frac{{d\theta }_{\text{rhizosphere}}}{{d\theta }_{\text{soil}}}$. This was done separately for transpiring and nontranspiring plants, as the mechanisms driving water movement differ between these two physiological states.

In transpiring plants, this ratio serves as an indicator of which compartment is more actively depleted and therefore contributes more to RWU. In nontranspiring plants, where no active uptake occurs, we assume water potential equilibrates throughout the soil, leading to similar drying rates in both compartments. To establish this baseline, we plotted rhizosphere water content against bulk soil water content over time for each nontranspiring sample and fit a linear regression. The slope of this regression represents the expected 1 : 1 drying trajectory without plant‐driven fluxes. This slope was then used as a reference to interpret deviations in transpiring plants, where higher slopes indicate preferential rhizosphere depletion due to active uptake. As nighttime transpiration could not be impeded, this reference light might not represent perfect equilibrium.

RWU was quantified by summing pixel intensities from the entire rhizobox in each neutron radiograph and converting them to total water content using pixel area. Since the soil surface was sealed (see Fig. S2), all water loss from the rhizobox was attributed to transpiration, making RWU a direct proxy for whole‐plant transpiration.

Although images were acquired every 30 s, water uptake was computed from differences between images separated by 15 min to optimize signal‐to‐noise ratio while retaining physiological temporal resolution and capturing meaningful physiological changes. This cadence captured the gradual changes in water distribution associated with transpiration while minimizing noise from detector fluctuations and short‐term plant movements. The time series was smoothed using a Savitzky–Golay filter (Savitzky & Golay, 1965), and RWU rates were calculated as the change in water content over each 15‐min interval. These rates were then averaged across the full transpiration period of each plant, which ranged from 4 to 10 h, yielding a single representative value of whole‐plant uptake. In addition, stomatal conductance was measured for some of the plants (*n* = 5 per type of soil) to test how well RWU scaled with leaf‐level transpiration rates.

## Results

**Fig. 3:**
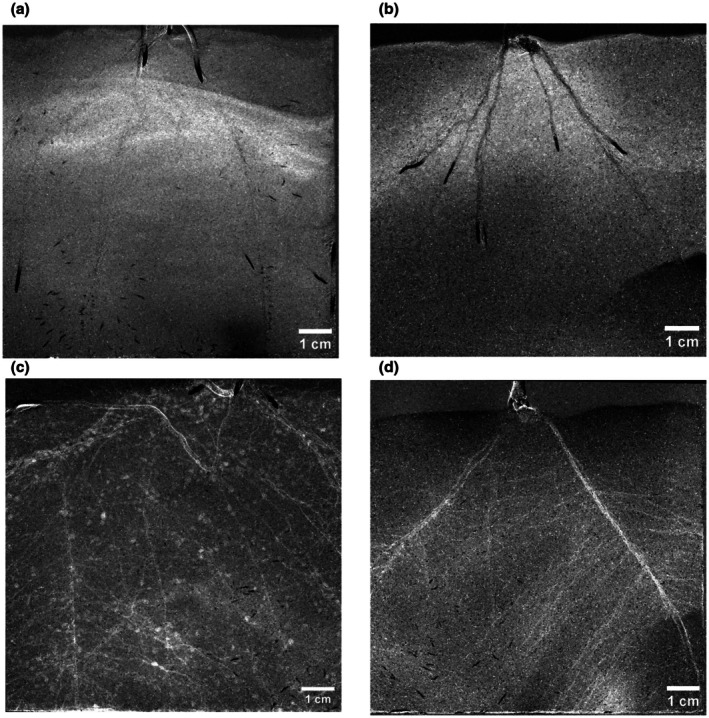
Spatial changes in water distribution are shown by differences between the wettest and driest images for sand (left column) and loam (right column). Bright pixels represent water loss, and dark pixels represent water gain (in this case, indicating root growth). (a, b) show that under wet conditions, water depletion occurs mainly in the bulk soil, while (c, d) show that under drier conditions, water depletion shifts primarily to the rhizosphere. Bars, 1 cm. Pixel size *c*. 70 μm (2048 × 2048 pixels covering a 150 × 150 mm field of view). Images show *Zea mays* L. roots.

Neutron radiography reveals distinct patterns of RWU depending on soil moisture conditions (Fig. 3): Under wet conditions, water was predominantly extracted from the bulk soil, as indicated by decreased moisture levels in the soil matrix (a, b), where lighter pixels represent zones of water depletion. By contrast, under drier conditions, water uptake shifted toward the rhizosphere (c, d). This is indicated by lighter pixels that correspond to the root zone – signaling that water is being extracted directly from the rhizosphere and root tissues.

**Fig. 4:**
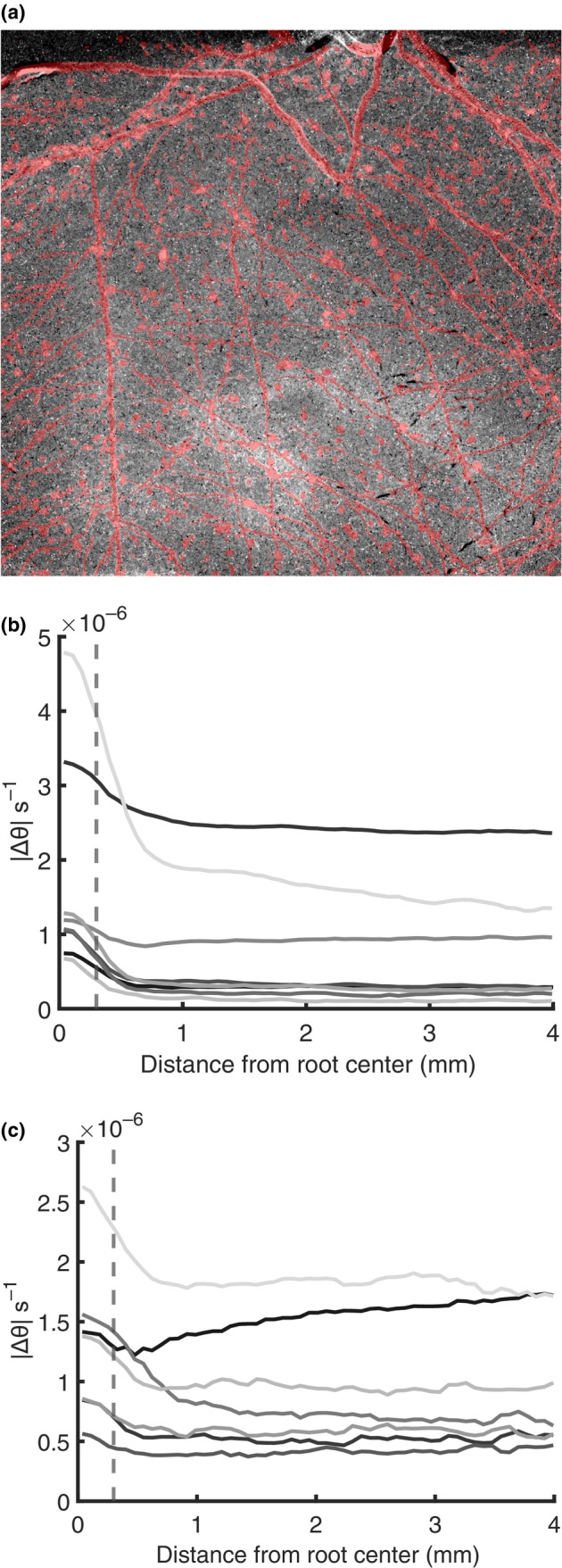
Spatial characterization of water depletion in dry conditions in *Zea mays* L. (a) Spatial overlap between the segmented root system and the difference between the radiographs of the wettest and driest states of an exemplary sample. Brighter pixels indicate water loss, while darker pixels indicate water gain. Water depletion extends beyond the root boundaries, highlighting that drying affects not only the root but also the surrounding soil. Radial profile of water content changes over time ($\left|\Delta \theta s^{-1}\right|$) for sand (b) and loam (c), measured from the root centre outward to *c*. 4 mm. The steepest decline occurs within the first *c*. 1 mm. Given that the average root diameter is < 0.3 mm (dashed line), this indicates that most water depletion is localized to the root–rhizosphere region, supporting our interpretation of this domain as a single functional unit rather than separating root and soil contributions. Different colors indicate different samples.

Following the qualitative visualization of differential water use, we quantified temporal dynamics of water depletion in the rhizosphere and bulk soil. We calculated the average water content far from the root ($\theta_{\mathrm{soil}}$) and in the root zone, including the rhizosphere ($\theta_{\text{rhizo}}$). Image analysis shows that water depletion extends *c*. 1 mm beyond the root surface – well past the root diameter (< 0.3 mm) – indicating that drying is not confined to the root tissues alone but includes surrounding soil (Fig. 4).

Detailed definitions and segmentation of the regions are explained in the Materials and Methods section (Fig. 2). Rather than trying to separate the interconnected root cortex and rhizosphere, we interpret the root cortex–rhizosphere region as a single functional unit, from here on referred to as the rhizosphere – defined as the root cortex and the immediately adjacent soil captured within the *c*. 1 cm projection path.

Water content changes in bulk soil and rhizosphere showed a clear dependence on soil water potential, and such dependence differed between soil types. The relative contribution of rhizosphere and bulk soil to RWU, expressed as $\frac{d\theta_{\text{rhizosphere}}}{d\theta_{\mathrm{soil}}}$ ratio, varied with soil water potential. Higher values indicate preferential water depletion from the rhizosphere, root hairs and root zone.

**Fig. 5:**
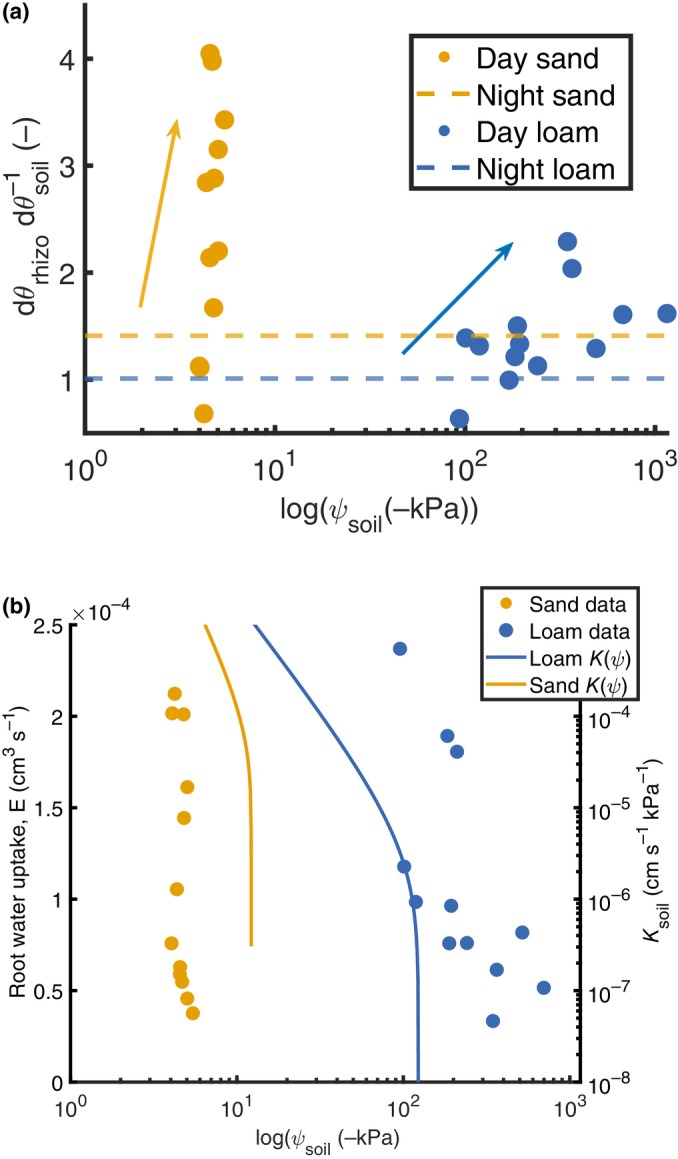
Spatiotemporal water dynamics and root water uptake under drying soil conditions in *Zea mays* L. (a) Ratio of water content changes ($\frac{{d\theta }_{\text{rhizosphere}}}{{d\theta }_{\text{bulk}}}$) in the rhizosphere relative to the bulk soil, indicating higher water depletion of the rhizosphere when the ratio increases. Arrows indicate the direction of soil drying. The dashed line marks the nighttime average ratio when water potential equilibrates. (b) Root water uptake as a function of soil water potential, illustrating decreasing uptake at more negative soil water potential that is dry soil. Solid lines indicate the corresponding soil hydraulic conductivity functions ($K\left(\psi \right)$) for sand and loam, illustrating how the decline in root water uptakes happens in concomitance with the drop in hydraulic conductivity.

The ratio $\frac{{d\theta }_{\text{rhizosphere}}}{{d\theta }_{\text{soil}}}$ increased with declining soil water potential (Fig. 5a). The dotted line represents the average $\frac{{d\theta }_{\text{rhizosphere}}}{{d\theta }_{\text{soil}}}$ at nighttime, when rhizosphere and bulk soil approach the same water potential and differences in water content reflect only their distinct water retention properties. A shift toward rhizosphere‐dominated uptake occurs when the ratio exceeds this nighttime baseline, signaling active depletion of rhizosphere water as the soil dries. Measurements of RWU at night showed a small but nonzero flux, indicating that nocturnal transpiration was minimal but not absent. This means the nighttime baseline might be slightly higher than it would be under full equilibrium, so the reported shift toward rhizosphere depletion is a conservative estimate. This transition is strongly texture‐dependent. In sandy soil, rhizosphere depletion emerges *c*. −5 kPa, indicative of a narrow soil operating range before significant hydraulic limitation occurs. By contrast, loamy soils exhibit this transition near −200 kPa, extending over a broader potential range, consistent with greater water retention capacity.

These values should be regarded as indicative ranges rather than precise thresholds, as the transition is gradual and reflects the onset of divergence between rhizosphere and bulk soil water dynamics, emerging from the combined dataset rather than a discrete change‐point. Soil water potential was derived from soil water contents obtained from the images, using the respective soil water retention curves reported in Vetterlein *et al*. (2021) to convert volumetric water content to water potential.

Fig. 5(b) further contextualizes these dynamics by depicting RWU as a function of soil water potential alongside modeled soil hydraulic conductivity curves ($K\left(\psi \right)$) for sand and loam (solid lines). In sandy soils, RWU declines steeply with decreasing soil water potential, coinciding with a rapid drop in hydraulic conductivity, while water uptake in loamy soils is sustained for more negative soil water potentials. The onset of water uptake decline occurs at significantly different thresholds: sharply at −5 kPa in sand, compared with a gradual decline from −100 to −1000 kPa in loam, as expected from the theory.

**Fig. 6:**
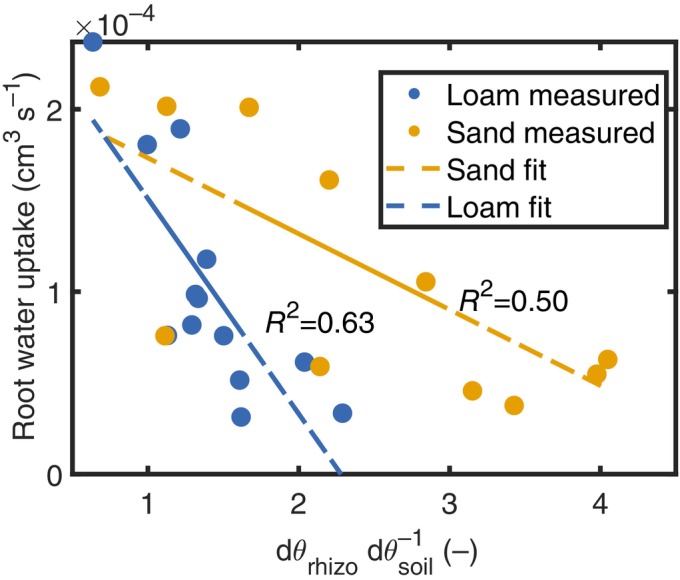
Increase in the relative rate of water depletion in the rhizosphere compared with the bulk soil in *Zea mays* L. ($\frac{d\theta_{\text{rhizosphere}}}{d\theta_{\text{soil}}}$) indicates a shift toward depletion of rhizosphere soil moisture. This shift is associated with reduced root water uptake, suggesting that under drought stress, the limited water volume in the rhizosphere alone cannot sustain transpiration demands.

The relationship between rhizosphere depletion and decline in water uptake is illustrated in Fig. 6. As the ratio $\frac{{d\theta }_{\text{rhizosphere}}}{{d\theta }_{\text{soil}}}$ increases – indicating a higher depletion in the rhizosphere – there is a concurrent reduction in total RWU. This inverse relationship highlights the regulatory influence of rhizosphere‐scale hydraulic properties on plant water status and transpiration during drought. As drying progresses, the water supply of the soil cannot match the water extraction from the root surface and water depletion becomes confined to the rhizosphere. However, the limited volume of the latter becomes rapidly insufficient to sustain transpiration, resulting in a sharp decline in water uptake.

To assess whether reductions in RWU correspond to a stomatal response, we compared RWU with stomatal conductance measured under comparable conditions. The strong correlation between the two (see Fig. S3) confirms that declines in RWU are tightly coupled with reduced stomatal conductance and how the observed decrease in RWU marks the initiation of stomatal regulation.

## Discussion

Water depletion in the rhizosphere has been long hypothesized as an indicator of water limitation to plants (Gardner, 1960; Passioura, 1980). However, direct observation for this process, particularly its spatial and temporal dynamics and its link to stomatal regulation, has remained elusive. We used high‐resolution time‐series neutron radiography to visualize water depletion near roots during soil drying. We found that water was depleted in the bulk soil in wet conditions. As drying progressed and the ability of the soil to sustain flow toward the root surface diminished, water depletion shifted to the rhizosphere, where enhanced water retention properties allowed water to persist longer. This shift was concomitant with a marked decline in transpiration.

The points at which the rate of water loss from the rhizosphere exceed that from the bulk soil fits with longtime established theories of RWU (Gardner, 1960) and align with the critical soil water potentials predicted from the soil hydraulic conductivity curve (Cai *et al*., 2021; Wankmüller *et al*., 2024). In sandy soils, where weak capillary forces lead to early hydraulic disconnection – when the bulk‐soil supply can no longer meet transpiration demand and water uptake becomes restricted to rhizosphere films and root tissues – we observed a rapid decline in RWU at relatively high‐water potentials (−5 to −20 kPa). This was accompanied by an early decrease in RWU. The transition to soil water limitation was more gradual in loam soils and it occurred at lower water potentials (−100 to −300 kPa), consistent with its less steep hydraulic conductivity curves. These results underscore the importance of soil physical properties in shaping the location and timing of hydraulic limitation.

As water uptake became increasingly confined to a small region (e.g. the rhizosphere), the water potential at the root surface is expected to decline at an increasing pace. The resulting fast drop in the rhizosphere water potential imposes a strong gradient along the soil–plant–atmosphere continuum, accelerating declines in xylem hydrostatic pressure and, consequently, in leaf water potential. Such decreases in xylem water potential can promote embolism formation and leaf dehydration (Sperry *et al*., 2002; Martin‐StPaul *et al*., 2017; Brodribb *et al*., 2021), which reduce internal plant hydraulic conductance and lead to a xylem cavitation feedback (Tonet *et al*., 2023). However, the observation that soil water limitation in sandy soils occurs at comparatively high‐water potentials indicates that hydraulic constraints in sandy soils emerged well before any substantial decline in plant hydraulic conductance. By contrast, in fine‐textured soils, decreases in soil and plant conductance appear more closely coupled, suggesting a coordinated reduction in hydraulic capacity along the soil–plant continuum. We argue that the accompanying decline in leaf water potential provides the immediate signal for stomatal closure, a response that does not discriminate between soil‐derived limitations to water flow and changes in plant hydraulic conductance, allowing stomata to close even when plant hydraulic capacity remains largely unaffected.

The shift from bulk soil to rhizosphere water uptake is not merely spatial but carries important functional consequences. The rhizosphere is the principal site of water and nutrient exchange and hosts complex biological interactions, including root hairs, microbial communities, and exudates that shape its hydraulic properties. Mucilage and exudates enhance rhizosphere hydraulic continuity by maintaining hydraulic connectivity as the soil dries (Carminati *et al*., 2010; Moradi *et al*., 2012), while root hairs expand the effective absorption surface and smooth water potential gradients (Carminati *et al*., 2017; Cai *et al*., 2021; Duddek *et al*., 2023). In combination with microbial EPS, which promote soil aggregation and water retention (Albalasmeh & Ghezzehei, 2014; Benard *et al*., 2019b; Kaniz *et al*., 2023), these features create a hydrated microenvironment around roots that can sustain water uptake during early drought. Anatomical features such as cortical lacunae may further act as transient water reservoirs (Cuneo *et al*., 2016), buffering internal hydraulic gradients. Consuming the water reservoir of the rhizosphere has additional negative effects besides the hydraulic ones, by limiting the diffusion of nutrients to plants and associated microorganisms. Therefore, using this water slowly has many advantages for plants.

The concept of the rhizosphere as a hydraulic buffer was first proposed by McCully & Boyer (1997), who studied the water holding capacity of root cap mucilage. They proposed that the functional role of mucilage in buffering drought stress should be minor, in part due to its limited volume. In our experiments, the additional water retained in the rhizosphere sustained transpiration for several tens of minutes (see Methods S1 for more details), the same timescale over which stomatal closure typically occurs. This temporal alignment suggests that the rhizosphere may function as a short‐term daily reservoir, preserving hydraulic continuity just long enough to stabilize leaf water potential and allow for the initiation of coordinated stomatal responses. Rather than serving as a passive remnant of prior moisture conditions, the rhizosphere may play an active and time‐sensitive role in mediating the transition from soil‐driven to plant‐regulated responses to drought.

The hydraulic function of the rhizosphere is likely to differ across plant functional types, shaped by variation in internal hydraulic capacitance and root architecture. Herbaceous annuals, with limited internal water storage, may rely heavily on rhizosphere‐retained moisture to maintain water uptake during rapid declines in soil moisture availability. By contrast, woody perennials can buffer transient imbalances through internal capacitance, yet once these storages are depleted – and if uptake remains restricted to a hydraulically disconnected rhizosphere – even trees may experience sharp declines in leaf water potential (McCarthy *et al*., 2025). Beyond capacitance, differences in root architecture – including root hair density, branching patterns, and overall root system connectivity – can also shift the threshold at which hydraulic limitation arises, by modifying the effective absorptive surface area and the continuity of the hydraulic contact between roots and soil. This scenario could explain why trees, despite their structural buffering capacity, often exhibit delayed but rapid declines in function under prolonged or repeated drought (Salomón *et al*., 2017; Preisler *et al*., 2022). These considerations point to the rhizosphere not just as a hydraulic interface, but as a dynamic regulator whose function is likely to be coordinated with other plant strategies, such as shallow and deep roots, or fast and slow growth.

While neutron imaging can, in principle, be affected by beam‐related artifacts, several factors indicate that the observed shift from bulk‐ to rhizosphere‐dominated uptake is not an imaging artifact. The neutron flux used was too low to induce detectable heating or water redistribution within the sample. The transition was reproduced across independent beamtime sessions and occurred over timescales far longer than the image acquisition interval, confirming that it reflects a genuine hydraulic process rather than a limitation of temporal or spatial resolution.

In summary, our findings highlight the rhizosphere not simply as the site of water uptake, but as the critical hydraulic interface where drought stress first manifests, particularly in coarse soils, such as sand. Acting both as an initial trigger that starts the drop in water potential that ultimately leads to stomatal closure and a transient reservoir that delays its onset, the rhizosphere emerges as a pivotal control point in early drought response – where targeted monitoring and management could meaningfully enhance plant resilience under water‐limited conditions.

## Competing interests

None declared.

## Author contributions

SDB, AC and PB designed the research. SDB, AC, PB, RJ and FJPW performed the research with support from SA and AK SDB, AC and PB analyzed the data. SDB and AC wrote the paper with contributions from all authors. AN and TJB provided critical input on the interpretation and discussion of the results.

## Disclaimer

The New Phytologist Foundation remains neutral with regard to jurisdictional claims in maps and in any institutional affiliations.

## Supporting information


**Fig. S1** Exemplary radiography scan.
**Fig. S2** Photograph of a rhizobox.
**Fig. S3** Root water uptake as a function of stomatal conductance for both sand and loam.
**Methods S1** Procedures for estimating rhizosphere water volume and depletion.
Please note: Wiley is not responsible for the content or functionality of any Supporting Information supplied by the authors. Any queries (other than missing material) should be directed to the *New Phytologist* Central Office.

## Data Availability

The data supporting the results of this study are available in the ETH Zürich Research Collection and can be accessed via the following link: https://doi.org/10.3929/ethz-c-000789374.
