# Supplementary material for: Early signals of water limitations begin at the root–soil interface: linking rhizosphere drying to water uptake decline
Source: New Phytol. 2025 Dec 26;249(6):2909–18. doi: 10.1111/nph.70879 (PMC12917449; doi:10.1111/nph.70879)
Supplement: Supplementary file 1 — Fig. S1 Exemplary radiography scan. Fig. S2 Photograph of a rhizobox. Fig. S3 Root water uptake as a function of stomatal conductance for both sand and loam. Methods S1 Procedures for estimating rhizosphere water volume and depletion. Please note: Wiley is not responsible for the content or functionality of any Supporting Information supplied by the authors. Any queries (other than missing material) should be directed to the New Phytologist Central Office. [file NPH-249-2909-s001.pdf]

New Phytologist Supporting Information

Article title: Initial stomatal closure begins at the root-soil interface: neutron radiography links rhizosphere drying to transpiration decline.
Authors: Sara Di Bert, Pascal Benard, Rong Jia, Fabian JP Wankmüller, Seren Azad, Anders Kaestner, Andrea Nardini, Timothy J. Brodribb and Andrea Carminati
Article acceptance date: 4 December 2025

The following Supporting Information is available for this article:

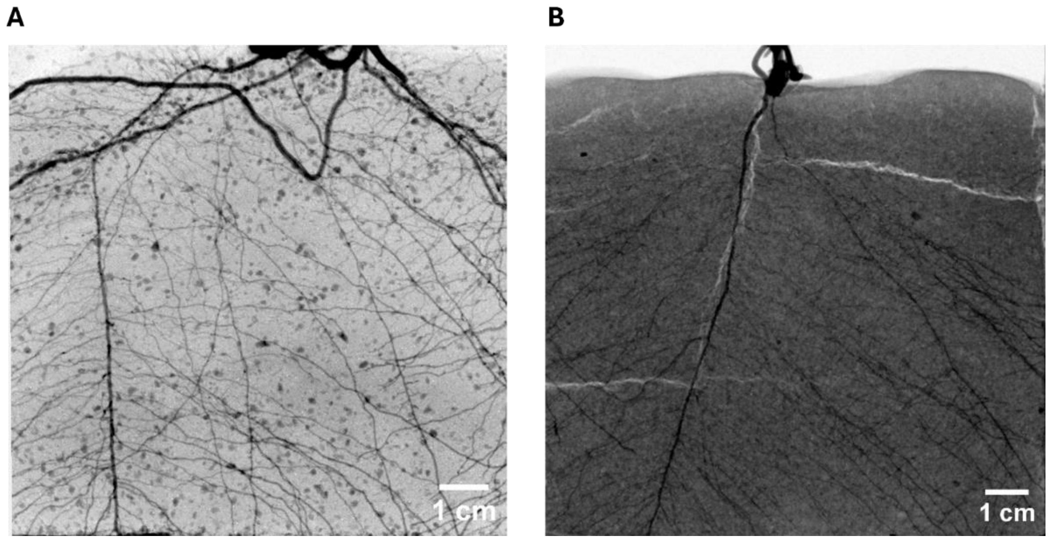

Fig. S1 Exemplary radiography scan of the 2-week-old maize (*Zea mays* L.) sample in sand (A) and loam (B). Grey value is proportional to the water content.

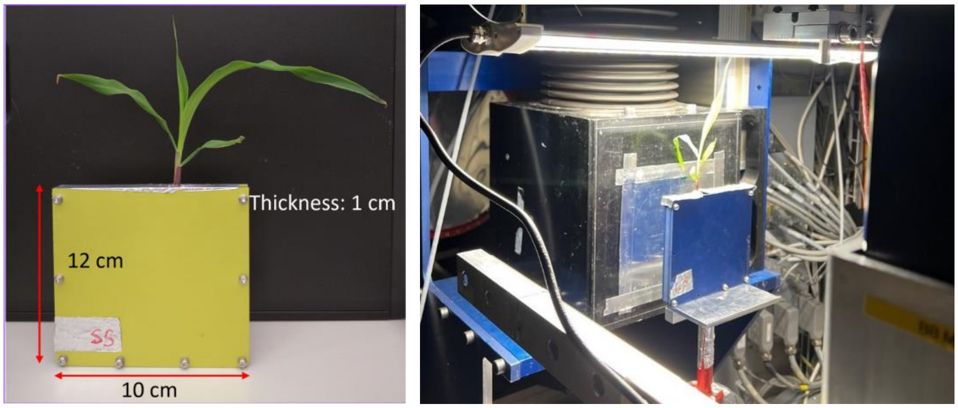

Fig. S2: Photograph of the rhizobox positioned at the neutron beamline. The soil surface was sealed with aluminium tape to prevent evaporation. Rhizobox dimensions: 10 × 12 × 1 cm.

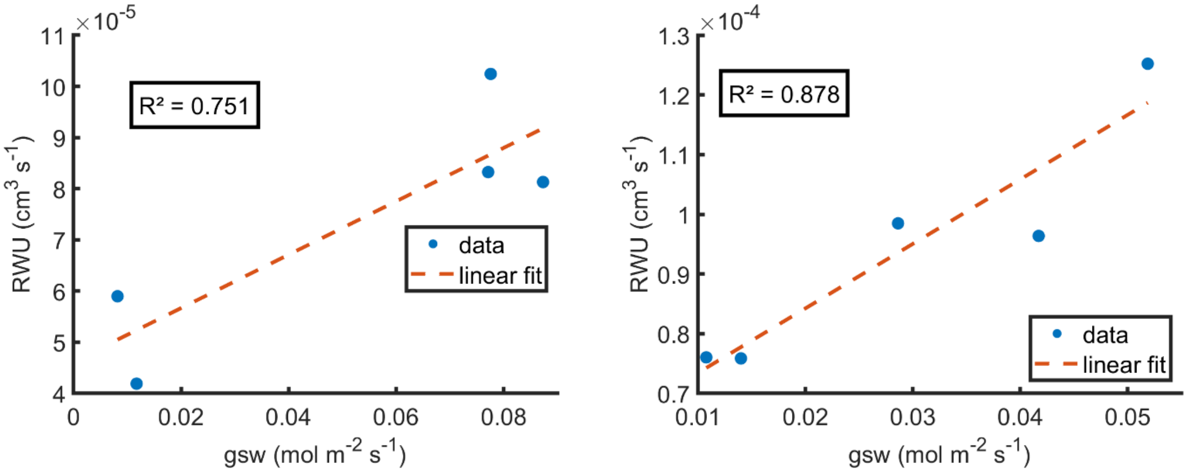

Fig. S3: Root water uptake (RWU) as a function of stomatal conductance (gsw) for sand (a) and loam (b). The strong positive correlations indicate that declines in root water uptake are closely associated with reductions in stomatal conductance, suggesting a tight coordination between belowground water uptake and aboveground gas exchange.

Methods S1: The volume of water retained in the rhizosphere and the time required for its depletion were estimated from segmented neutron radiographs. The rhizosphere was approximated as a cylindrical sleeve surrounding the root, with its volume calculated as $V_{rhizo=}\pi r^2 L$, where *L* is total root length and *r* the rhizosphere radius (estimated from the radiographs). The additional water stored in this region was obtained from the difference in volumetric water content between rhizosphere and bulk soil before and after transpiration (Δθ). The corresponding available water volume was

$$V_{w=} = \Delta\theta \times V_{rhizo}$$

and the depletion time as

$$t_{deplete} = \frac{V_w}{Q}$$

where *Q* is the average transpiration rate from total water loss in sealed rhizoboxes.
